# A Diagnostic Dilemma of Secondary Hemophagocytosis Lymphohistiocytosis in an Elderly Patient

**DOI:** 10.7759/cureus.8482

**Published:** 2020-06-07

**Authors:** Vani Mulkareddy, Varun Bhalla, Ankit Garg

**Affiliations:** 1 Internal Medicine, Rochester General Hospital, Rochester, USA; 2 Internal Medicine, Kasturba Medical College, Manipal, IND

**Keywords:** secondary hemophagocytic lymphohistiocytosis, elderly, diagnostic dilemma, fever of unknown origin

## Abstract

Hemophagocytic lymphohistiocytosis (HLH) is a rapidly progressive fatal condition. Although well described in the pediatric population, cases of secondary HLH are seen in adolescents and young adults. In the elderly, HLH has been shown to have a poor prognosis. Owing to its varied presentation and multisystemic involvement, diagnosis is often delayed. Due to its high mortality, prompt diagnosis and treatment are crucial. Here we present a case of secondary HLH in a 69-year-old male, who presented with fever for one week. Initial laboratory workup revealed a bicytopenia and elevated creatinine. He was initially treated with broad-spectrum antibiotics; however, a comprehensive infectious workup was negative. CT scan of the abdomen revealed splenomegaly. Further investigations revealed an elevated ferritin and triglycerides. Due to the constellation of findings, he was started on corticosteroids for concerns of HLH. Bone marrow biopsy was obtained, which revealed dysplastic changes and hemophagocytosis, consistent with HLH. This case highlights the diagnostic challenge and prognosis of HLH in the elderly population, suggesting that diagnosis and treatment should not be delayed for histological confirmation.

## Introduction

Hemophagocytic lymphohistiocytosis (HLH) is a fatal and rapidly progressive immunodeficiency, characterized by concurrent dysregulation of immune regulators and unremitted inflammation [1]. Although well described in the pediatric population as primary HLH, secondary cases have been reported in all age groups. Recently, an increasing number of HLH cases are being reported in the elderly. Initially described as a familial immunological disorder, later HLH was also recognized to present sporadically in association with infection, malignancy, and rheumatological disorders. HLH commonly presents as an acute febrile illness with multisystemic involvement. Laboratory findings, including hyperferritinemia, are often wide ranging and nonspecific. Timely diagnosis can be challenging due to the variable presentation, rarity of the disease, and laborious diagnosing testing. Prompt diagnosis and treatment are crucial due to its high mortality rate of 50%-75% [1]. Here we present a case of secondary HLH, presenting as fever of unknown origin with acute kidney injury in a 69-year-old gentleman. A comprehensive workup revealed splenomegaly, bicytopenia, hyperferritinemia, hypertriglyceridemia, and biopsy consistent with HLH. Although corticosteroids were started prior to histological confirmation, prognosis remained poor. This case highlights the importance of high clinical suspicion, prompt diagnosis, and early treatment of HLH in the elderly. 

## Case presentation

A 69-year-old gentleman with a past medical history of stage IV chronic kidney disease secondary to IgA nephropathy presented with fever and lethargy for one week. In the past one week, he had two mechanical falls associated with lightheadedness on standing. He had no history of loss of consciousness, head injury, sore throat, cough, abdominal pain, diarrhea, dysuria, or recent sick contacts. There was no significant family or social history. Examination was remarkable for a pulse of 103 beats/minute, blood pressure of 88/50 mmHg, temperature of 101.5°F, pallor, and no other systemic focal findings.

Laboratory workup was significant for creatinine of 5.2 mg/dL (baseline 3.6 mg/dL), platelet count of 60 x 10^3^/uL, white blood cell count of 6.4 x 10^3^/uL, hemoglobin of 9.7 g/dL (baseline 12.5 g/dL), and triglycerides of 260 mg/dL. He was initially started on broad-spectrum antibiotics with vancomycin, cefepime, and metronidazole.

**Figure 1:**
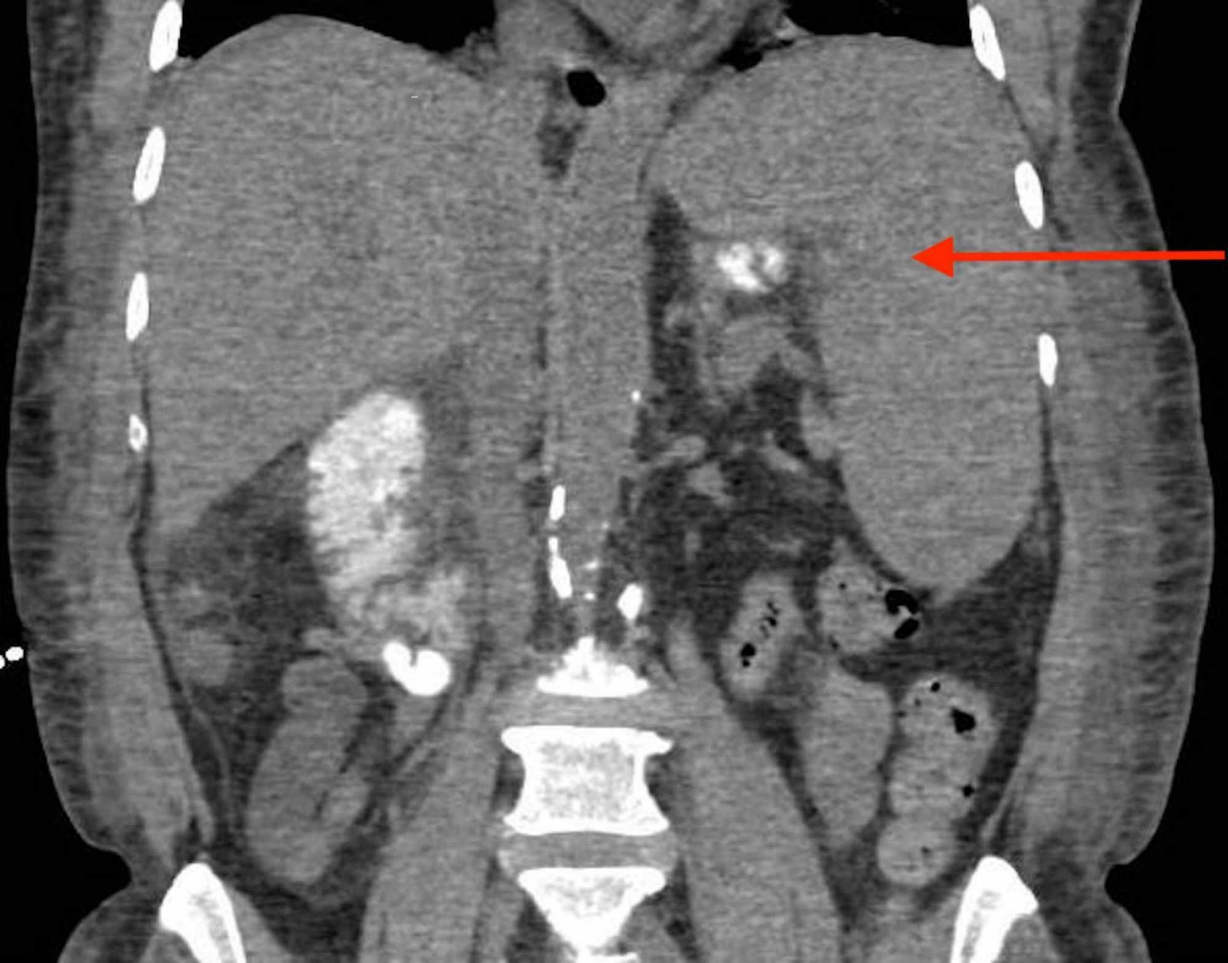
Coronal section of CT abdomen revealing splenomegaly of 17 cm (red arrow).

Initial infectious workup, including blood cultures, urine analysis, and chest x-ray, was negative. Further infectious workup with atypical/viral panel, Epstein-Barr viral capsid antigen antibodies, hepatitis viral panel, human immunodeficiency virus (HIV) antibodies, Mantoux test, acid fast bacilli cultures, fungal cultures, urine histoplasma antigen, and cytomegalovirus IgM antibody were all negative. Imaging including transthoracic echocardiogram and transesophageal echocardiogram showed no abnormalities. CT of the abdomen revealed splenomegaly of 17 centimeters (cm) (Figure 1). Antibiotics were then discontinued as infectious workup was negative.

**Figure 2:**
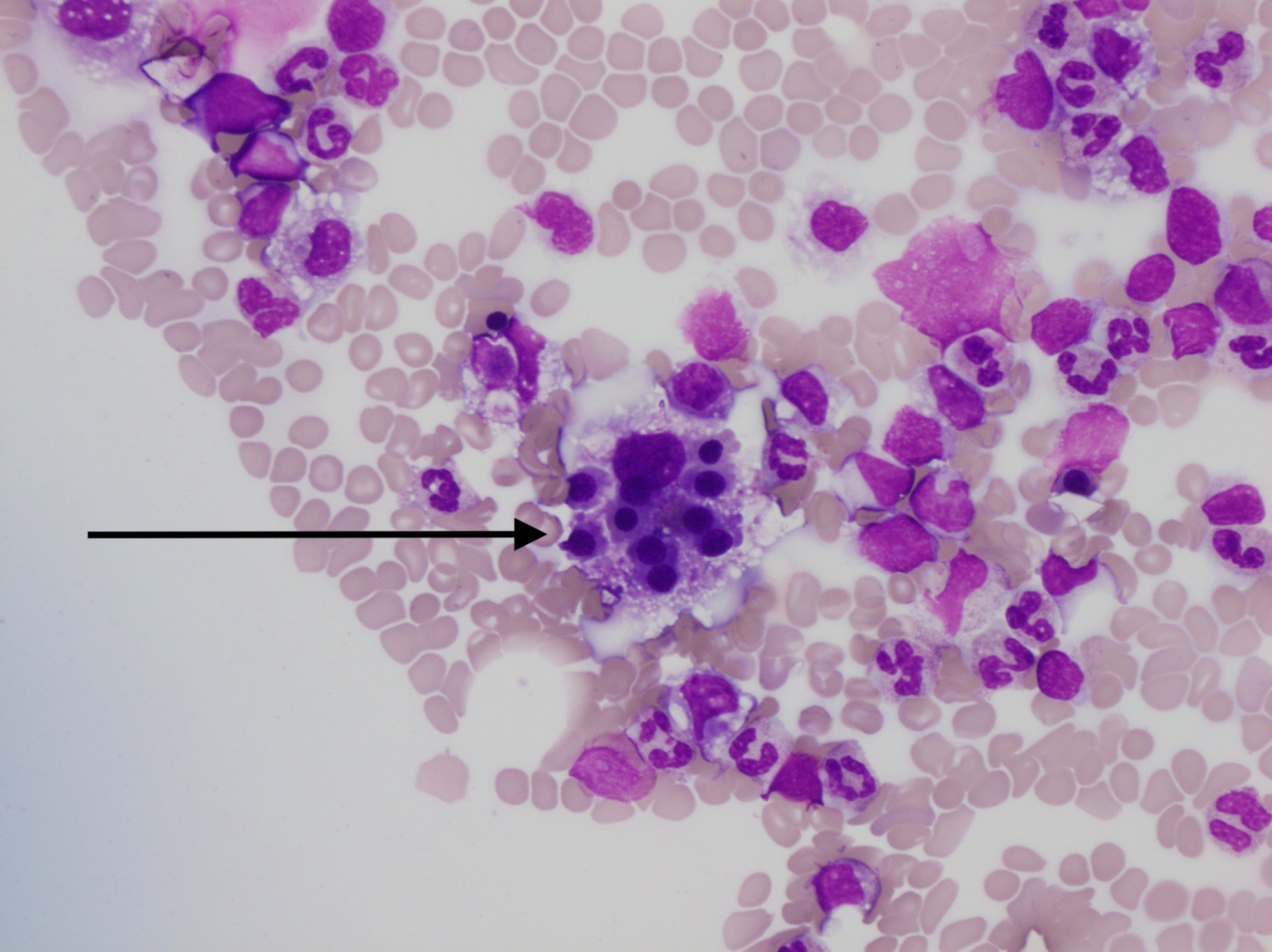
Pathology of bone marrow biopsy revealing hemophagocytosis of multiple nucleated red blood cells (black arrow).

Other causes of fever, including hematological and rheumatological etiologies, were investigated. Antinuclear antibody screen was negative. Ferritin was elevated at 2,857 mcg/L and lactate dehydrogenase (LDH) was elevated to 688 U/L. Peripheral smear showed nucleated red blood cells, atypical lymphoid cells, and no schistocytes, immature cells, or dysplastic appearing neutrophils. Flow cytometry was nondiagnostic. Due to splenomegaly, fever, elevated ferritin, and cytopenia, he was started on dexamethasone 20 mg for concern of HLH. He continued to have worsening thrombocytopenia and renal failure. Acute kidney injury was thought to be secondary to acute tubular necrosis. Bone marrow biopsy was obtained, which revealed dysplastic changes and hemophagocytosis (Figure 2). Despite treatment for a week, the patient passed away from hyperkalemia-induced cardiac arrest. 

## Discussion

HLH is a life-threatening, primary immunodeficiency, causing homeostatic dysregulation of the immune system. It is characterized by an unchecked activation of cluster of differentiation (CD) 8+ T-lymphocytes and macrophages resulting in widespread organ damage, most notably in the liver, bone marrow, and central nervous system [1]. This syndrome results from a broad set of inherited or induced triggers, most of which impair cytotoxic T-cell and natural killer (NK) cell function. This results in failure to eliminate damaged or infected cells, including macrophages, resulting in excessive cytokine secretion and tissue damage. The concurrent dysregulation of immune regulators and unremitted inflammation differentiates HLH from other inflammatory and immunodeficiency states [1].

HLH is categorized into primary and secondary forms based on etiology. Familial genetic defect within the perforin-mediated cytotoxicity pathway is the hallmark of primary HLH [2]. Classically seen in the pediatric population, it has an incidence of one in 3,000 inpatient admissions [1,3]. Although HLH is well studied in this younger cohort, cases are increasingly being reported in young adults and now in the elderly. Secondary or acquired HLH is seen without familial immunodeficiencies and has clear triggers for developing an acute illness. Infections, most commonly Ebstein-Barr virus, malignancy including lymphomas, and autoimmune disorders, are a few of the known inducers of HLH. In our patient, no clear trigger was identified. However, there is emerging evidence showing that a subset of secondary HLH may have a heterozygous genetic mutation, resulting in partial gene expression. It commonly affects adolescent and adult populations with an incidence of one in 800,000 persons [3]. Demographically it has a male predominance with an ethnic predisposition for malignancy-associated HLH in Japanese and East Asians [4,5].

The clinical presentation of HLH has puzzled clinicians with its broad and varied manifestations. HLH can affect any organ system leading to respiratory abnormalities, renal dysfunctions, skin manifestations, and circulatory disturbances. It commonly presents as a febrile illness with multiple organ dysfunction mimicking infections, malignancies, and autoimmune disorders. In a study of 71 elderly patients with HLH, fever was the presenting symptom in all 100% of patients [6]. The HLH-2004 study included 369 cases, and fever was a presenting symptom in 95% of patients, while bicytopenia and splenomegaly were seen in 92% and 89% of cases, respectively [7].

Given the rarity and heterogeneous presentation, HLH is a challenge to diagnose clinically and pathologically. The diagnostic workup is similar in all age groups and should include workup for bone marrow insufficiency, liver abnormality, neurological involvement, and immune activation. Previously, elevated ferritin was thought to be pathognomonic of HLH. In the pediatric setting, hyperferritinemia of greater than 10,000 mcg/L has a specificity of 96% for HLH [8]. In contrast, elevated ferritin is not specific for HLH in adult age groups. A retrospective study on adults with ferritin greater than 50,000 mcg/L found that hyperferritinemia was most commonly observed in patients with renal failure, hepatocellular injury, infections, and hematological malignancies. Only 19% of patients in the cohort studied had HLH [9]. Workup of HLH should include bone marrow biopsy to assess the cause of cytopenias and to identify hemophagocytosis. Bone marrow aspirate should ideally be cultured and investigated to rule out infectious and malignant differentials. Histologically, bone marrow cellularity can vary [1]. Hemophagocytosis is the only histomorphological criterion, but it is neither pathognomonic nor diagnostic of HLH. Although hemophagocytosis is reported in a wide range of 25% to 100% of cases, phagocytosis of nucleated cells, especially multiple nucleated cells, strongly correlates with HLH [10,11]. While objective thresholds for clinical and laboratory findings are clearly defined, there is no evidence-based guideline for reporting findings nor a well-defined quantitative threshold to pathologically diagnose hemophagocytosis in the bone marrow.

The constellation of clinical and laboratory abnormalities can be grouped together to make a composite diagnostic criteria, Histiocyte Society HLH-2004 diagnostic criteria [1]. Although the criteria was initially designed to be used in clinical trials, it is an appropriate diagnostic tool to help accurately identify patients with HLH. To fulfill the criteria, patients need to either have a molecular gene defect consistent with HLH or have five out of eight clinical criteria. The eight criteria include fever, splenomegaly, peripheral blood cytopenia affecting at least two cell lineages, elevated ferritin more than 500 mcg/L, hypertriglyceridemia and/or hypofibrinogenemia, hemophagocytosis (in the bone marrow, spleen, or lymph nodes), low or absent NK cell activity, or elevated soluble CD25 [12]. The patient reported above met six out of the eight criteria, including fever, splenomegaly, cytopenia, hypertriglyceridemia, hyperferritinemia, and hemophagocytosis. This suggests that diagnosis can be made without a biopsy or genetic testing, and treatment should not be delayed for further testing. It should also be noted that as this criterion was designed for clinical trials, it does not capture every HLH case. Thus, a high clinical suspicion is warranted.

Once suspected, expedient evaluation is important as early intervention is key in improving outcomes in these critically ill patients. Treatment with immunosuppression can significantly improve chances of survival. Mortality is high during the initial weeks of the disease due to multiorgan failure. Delay in treatment can be fatal, especially in cases of secondary HLH where mortality can be as high as 75% in adults [1]. Survival in familial HLH without treatment is limited to months [1]. Although most elderly cases present critically ill and require intensive care, mortality in elderly is not well studied. Prognosis has been shown to be worse in elderly patients, as well as those with neurological involvement, higher serum ferritin levels, thrombocytopenia, idiopathic, and malignant causes [8].

## Conclusions

Secondary HLH, although a rare disease in the elderly, has a grave prognosis. In patients with fever of unknown origin, HLH should be kept as a differential early on in the course as diagnostic and treatment delay is life threatening. As in this case, treatment should not be delayed for pathological confirmation, as early intervention improves prognosis. 
